# Antihypertensive medication adherence and associated factors among adult hypertensive patients at public hospitals in eastern Ethiopia: A Cross-sectional study

**DOI:** 10.1371/journal.pone.0322655

**Published:** 2025-05-28

**Authors:** Jemal Yousuf, Kedir Teji Roba, Nesredin Ahmed, Tefera Belsty, Fenta Wondimneh, Lema Daba, Tilahun Teshager, Indeshaw Ketema

**Affiliations:** 1 Department of Nursing, College of Medicine and Health Sciences, Dire Dawa University, Dire Dawa, Ethiopia; 2 School of Nursing, College of Health and Medical Sciences, Haramaya University, Harar, Ethiopia; 3 Department of Human Anatomy, School of Medicine, College of Health and Medical Sciences, Haramaya University, Harar, Ethiopia; 4 Department of Emergency and Critical Care Nursing, College of Health and Medical Sciences, Haramaya University, Harar, Ethiopia; Kwame Nkrumah University of Science and Technology College of Health Sciences, GHANA

## Abstract

**Background:**

Blood pressure regulation depends heavily on adherence to antihypertensive medication. Additionally, poor adherence to antihypertensive drugs leads to the development of hypertensive complications. However, little is knowen about the factors affecting antihypertensive medication adherance in Ethiopia, and no study has been conducted in the study settings. Therefore, this study aimed to assess antihypertensive medication adherence and associated factors among adult hypertensive patients in selected public hospitals in East Hararghe Zone, Eastern Ethiopia.

**Methods:**

A facility-based quantitative cross-sectional study was conducted from August 20 to September 20, 2023, among 364 adult hypertensive patients on follow-up in selected public hospitals of eastern Ethiopia. A simple random sampling method was used to select the study participants. Data were collected through face-to-face interviews using a pretested structured questionnaire. Drug adherence status was assessed using Morisky Medication Adherence Scale-8. Data was analyzed using Epi-Data 3.1 and STATA 17.0, applying bivariate and multivariate logistic regression techniques. The association was declared using p < 0.05.

**Results:**

The overall level of adherence to antihypertensive medications was 59.94% (95% CI: 54.65–65.06). Urban residence (AOR = 1.96; 95% CI: 1.21–3.18), college and higher education level (AOR = 3.41; 95% CI: 1.69–6.87), health insurance coverage user (AOR = 2.00; 95% CI: 1.11–3.59), having knowledge about hypertension (AOR = 1.75; 95% CI: 1.03–2.97), distance to health care facility less than 10 kilometers (AOR = 4.6; 95% CI: 1.97–10.73), having social support (AOR = 1.86; 95% CI: 1.13–3.08), and taking three and above medications (AOR = 0.28; 95% CI: 0.12–0.64) showed a statistically significant association with medication adherence.

**Conclusion:**

Adherence to antihypertensive medication was found to be low. This study identified place of residence, educational status, health insurance coverage, social support, knowledge of hypertension, distance from a health care facility, and number of medications as independent predictors of medication adherence. Therefore, improving accessibility of health care facilities, strengthening health insurance coverage, and providing health education about hypertension will improve antihypertensive medication adherence.

## Introduction

Hypertension (HTN), is a leading cause of morbidity globally, and adherence to antihypertensive therapy remains suboptimal, particularly in low-resource settings like Ethiopia [1].

Uncontrolled hypertension poses a serious public health risk to hypertensive patients in both high- and low-income countries [2,3]. The prevalence of uncontrolled BP has been higher in SSA than in Western countries in recent decades, meaning that three-quarters of hypertensive patients live with uncontrolled HTN [4,5]. The prevalence of uncontrolled HTN in Ethiopia is 48% [6]. Several factors have been identified that contribute to uncontrolled hypertension, including smoking, excessive alcohol consumption, salt consumption, obesity, and non-adherence to anti-hypertensive medications [7,8].

According to the World Health Organization (WHO), adherence is the extent to which a person follows the recommendations of medical or health professionals regarding medication administration and healthy lifestyle choices [9,10]. Numerous studies have shown that a variety of factors, including the kinds of medications prescribed, the relationship between patient and health care provider, the cost of the medications, forgetfulness, a lack of quality medication and health care services, and a lack of social support, particularly among elderly adults, affect adherence to antihypertensive medications [11,12].

Up to 40% to 50% of patients prescribed medications to treat chronic diseases, including hypertension and diabetes mellitus, fail to adhere to their prescribed medications [13]. Poor medication adherence account for two-thirds of uncontrolled HTN [14,15]. It leads to an increased incidence of complications, including coronary artery disease, acute myocardial infarction, peripheral vascular disease, stroke, congestive heart failure, and renal failure [16]. Additionally, it compromises the efforts of health care facilities, health care professionals, and policymakers to enhance and modify the quality of the health care of the people [17].

In Ethiopia, studies have shown that a significant proportion of patients do not take their prescribed antihypertensive medications, which negatively impacts medication effectiveness and the health care system [18–21]. Although several studies have been conducted worldwide on the effects of non-adherence to antihypertensive medications, the contributing factors are still unclear [22]. The few studies conducted in Ethiopia to assess the factors associated with adherence to antihypertensive medication have shown inconsistent results, and no research has been done in the area of interest [23]. Sociodemographic characteristics have a major impact on patient disease management, and the majority of research is conducted at single institutions; Little is known about the independent factors that influence adherence to antihypertensive medications. However, it is important to understand adherence to antihypertensive medication early to effectively manage symptoms and avoid potential complications. Accurate information about the incidence of adherence to antihypertensive medications is critical for adapting care policies and practices to improve disease control. Therefore, the aim of this study was to assess antihypertensive medication adherence and associated factors among adult hypertensive patients in selected public hospitals in East Hararghe zone, eastern Ethiopia.

## Methods and materials

### Study settings and period

The study was conducted from August 20 to September 20, 2023, in selected public hospitals in the East Hararghe zone, eastern Ethiopia. East Hararghe Zone is one of the nineteen zones of Oromia Regional State in eastern Ethiopia. The zone is 532 km from Addis Ababa, the capital city of Ethiopia and has an estimated total population of 3,066,150. The zone has 7 public hospitals (4 general hospitals and 3 primary hospitals) and 121 health centers. The study was conducted in three randomly selected public hospitals (by lottery methods) found in the East Hararghe zone, namely Dader General Hospital, Haramaya General Hospital, and Chelenko Primary Hospital. All hospitals are providing inpatient, outpatient, emergency, and delivery services for patients from surrounding catchment areas (Source: East Hararghe zonal health bureau, 2022). A total of 1020 adult hypertensive patients were on follow-up in selected public hospitals: 432 at Dader General Hospital, 456 at Haramaya General Hospital, and 132 at Chelenko Primary Hospital (Source: HMIS of selected hospitals).

### Study design and populations

A facility-based cross-sectional study design was conducted to determine antihypertensive medication adherence and associated factors among adult hypertensive patients in selected public hospitals in East Hararghe Zone, eastern Ethiopia. All hypertensive patients whose age was 18 years or older and had taken antihypertensive medication for at least six months before the survey in selected public hospitals were included in the study. Hypertensive patients with cognitive impairment and seriously ill patients who were unable to finish the interviews were excluded.

### Sample size determination

The sample size was determined using a single population proportion formula n= (z (α/2))^2^ p (1-p)/d^2^ by considering the following assumption: p = 31.4% adherence rate to antihypertensive medication from a previous study conducted at Nedjo General Hospital among hypertensive patients in chronic follow-up [24], z (α/2) = 1.96 (a 95% confidence interval), d = 0.05 (a 5% margin of error), and a 10% non-response rate, yielding a sample size of 364.

### Sampling techniques and procedures

Initially, three public hospitals (Dader General Hospital, Haramaya General Hospital, and Chelenko Primary Hospital) were randomly selected (by the lottery method) from seven public hospitals found in East Hararghe Zone. Then the sample size was proportionally allocated to each selected public hospital based on the number of each hospital’s annual hypertensive patients on follow-up. Specifically, Dader General Hospital, with 432 annual hypertensive patients, had a sample size of 154; Haramaya Hospital, with 456 hypertensive patients, had a sample size of 163; Chelenko Hospital, with 132 hypertensive patients, had a sample size of 47. Finally, the study participants were selected from each hospital using a simple random sampling technique.

### Data collection tools and methods

Data were collected using a pretested and structured questionnaire adopted from relevant literature [14,18,25–29] and some standard guidelines [30]. To maintain validity of the data collection tool, the questionnaire was first prepared in English, translated into local languages (Afan Oromo and Amharic), and then back-translated to English by language experts to check consistency. The tool consists of 6 parts: socio-demographic factors that contain 11 items, clinical factors with 11 items, personal factors with 13 items, organizational factors with 9 items, social support factors with 12 items, and tools for assessing antihypertensive medication adherence status containing 8 items. Data were collected by trained data collectors (9 BSc nurses) and supervisors (3 MSc nurses) using a structured and pre-tested interviewer-administered questionnaire through a face-to-face interview and medical record review.

This study utilizes the Morisky Medication Adherence Scale (MMAS-8) and the Duke Social Support and Stress Scale. These instruments were tested and validated using previous research to ensure they are suitable for the target population and environment, making it easier to compare the results with other studies.

The study participant’s antihypertensive medication adherence status was assessed using eight items of the Morisky Medication Adherence Scale (MMAS-8). A scoring scheme of “yes” = 1 and “no” = 0 was used for the first seven questions, the last question is a five-point Likert response with the options “never”, “once”, “sometimes”, “usually”, and “always”. The respondent who scored < 6 on the scale was considered non-adherent, and those who scored ≥ 6 were considered adherent [31].

The Duke social support and stress scale, which contains 12 items, was used to assess social support gained from family, friends, or significant others. The responses were coded as follows: “none” = 0, “some” = 1, “a lot” = 2, “yes” = 2, “no” = 0, and “there was no such person” = 0. The level of social support was considered “good” if they scored above or equal to the mean value of the scale; otherwise, it was considered “poor” social support [32].

### Variables

The outcome variable for this study was adherence to antihypertensive medications. Independent variables include socio-demographic factors (age, sex, religion, marital status, educational level, number of children, occupation, monthly income, residence, and family history of hypertension), clinical factors (co-morbidity, BP status, BMI, duration of diagnosis, duration of treatment, and number of antihypertensive drugs), personal factors (knowledge about antihypertensive medication and disease, alcohol consumption, smoking, and physical activity), organizational factors (health care provider-patient relationships, insurance coverage user, drug availability in the hospital, distance from the health care facility, and antihypertensive drugs changed by physician),and social support.

### Operational definitions

**Adherent:** respondents who scored ≥6 on Morisky’s medication adherence scale-8 [19].

**Non-adherent:** respondents who scored <6 in MMAS-8 [19].

**Knowledge:** respondents who scored the mean and above the mean score of knowledge related questions were considered knowledgeable [33].

**Co-morbidities:** respondents with one or more medical conditions in addition to hypertensions [34].

**Controlled hypertension:** refers to a BP measurement <140/90 mmHg for patients less than 60 years old; and <150/90 mmHg in patients aged 60 years and above [35].

**Uncontrolled hypertension:** refer to when the BP measurement is ≥ 140/90 mmHg and ≥130/80 mmHg for hypertensive patients with diabetic mellitus and chronic kidney disease [35].

**Active physical activity:** as per the recommendation of WHO, physical activity is considered “active” when at least 30 minutes of regular, moderate-intensity physical effort are performed for at least five days a week, totaling 150 minutes per week [36].

**Alcohol use:** participants who adhere to the JNC7 recommendations were deemed to be alcohol abstinent. Participants who reported that they didn’t drink alcohol at all or not drink any alcohol in the last 7 days were considered abstainers [29].

**Smoking:** respondents who reported 0 days of smoking in the last 7 days were considered non-smokers [29].

### Data quality control

To ensure high data quality, a structured questionnaire was adapted for the study. Data collectors and supervisors received a two day training on the study’s goals, the questionnaire, interviewing techniques, and data protection. A pretest was conducted on 5% of the sample size at Hiwot Fana Comprehensive Specialized Hospital to identify potential issues. Feedback from this pretest was used to refine the tool. During data collection, supervisors and the principal investigator reviewed the data daily for accuracy and completeness. Additionally, double data entry was implemented to cross-check information and resolve any discrepancies between clerks.

### Data processing and analysis

The collected data were coded, entered into Epi Data version 3.1, and exported to STATA version 17.0 software for cleaning and analysis. The missing values, outliers, and consistencies were checked by running frequency distributions. All variables were considered for bivariable logistic regression analysis. A covariate with a P-value less than 0.25 was further analyzed in a multivariable logistic regression analysis. Both crude and adjusted odds ratios were calculated with a 95% confidence interval to measure the strength of the association between the outcome and independent variables, and the variables with a P-value of less than 0.05 in the multivariable logistic analysis were used to declare a significant association. Model fitness was tested by the Hosmer-Lemeshow goodness of fit statistics and the model was adequately fitted with a p-value > 0.05.

### Ethical considerations

The proposal was approved by the Institutional Health Research Ethics Committee (IHREC) of Haramaya University College of Health and Medical Sciences under reference number IHREC/052/2023 and informed consent was obtained from participants to ensure data confidentiality and intended purpose.

## Results

### Socio-demographic characteristics of study participants

A total of 357 study participants were interviewed, achieving a response rate of 98.1%. The mean age (±SD) of the respondents was 46.19 (±11.40) years. Among the respondents, more than half (57.14%) were males, 58.82% were in the age group of 40–59 years, and the majority (73.67%) were Muslim followers. Half (50.42%) of the respondents were rural residents, and 81.79% of them were ever married. One hundred forty-six (40.9%) reported that they were farmers, while 33.61% were merchants. Regarding educational status, 31.65% had no formal education. More than half of the study participants (61.34%) had no family history of hypertension (Table 1).

**Table 1:** Socio-demographic characteristics of the study participants in selected public hospitals in East Hararghe zone, Eastern Ethiopia, 2023 (n = 357).

Variables	Category	Frequency (N)	Percentage (%)
Sex	Male	204	57.14
	Female	153	42.86
Age (in years)	18-39	84	23.53
	40-59	210	58.82
	60 and above	63	17.65
Marital status	Married	292	81.79
	Single	41	11.48
	Divorced	17	4.76
	Widowed	7	1.96
Educational level	No formal education	113	31.65
	Primary (grades 1–8)	80	22.42
	Secondary (grades 9–12)	72	20.17
	College and above	92	25.77
Occupation	Government employee	34	9.52
	Farmer	146	40.90
	Merchant	120	33.61
	Daily labor	20	5.60
	Others ^a^	37	10.36
Religion	Muslim	263	73.67
	Orthodox	60	16.81
	Protestant	32	8.96
	Others ^b^	2	0.56
Place of residence	Rural	180	50.42
	Urban	177	49.58
Monthly income (Eth. Birr)	<1000	78	21.85
	1000-5000	119	33.33
	>5000	160	44.82
Family history of HTN	Yes	138	38.66
	No	219	61.34
Number of children	<5	191	53.50
	≥5	166	46.50

^a^ Private employee, student, housewife.

^b^ Waqefata, catholic.

### Clinical and medication-related characteristics of hypertensive patients

The majority of respondents (89.08%) had hypertension for less than five years, 42.86% took one drug daily, and 27.17% of respondents had co morbidities. Diabetes mellitus was the most commonly reported co-morbidity, accounting for 20.45% of cases (n = 73). The majority of respondents (85.43%) reported that they were on treatment for less than five years. Regarding BMI, 45.1% of respondents were overweight (Table 2).

**Table 2:** Clinical and medication-related characteristics of adult hypertensive patients in selected public hospitals in East Hararghe zone, Eastern Ethiopia, 2023 (n = 357).

Variables	Category	Frequency (N)	Percentage (%)
BMI (in kg/m^2^)	<18.5	19	5.32
	18.5-24.9	155	43.42
	25.0-29.9	161	45.10
	≥30	22	6.16
Duration of HTN (in years)	<5	318	89.08
	5-10	32	8.96
	>10	7	1.96
Duration of treatment (in years)	<5	305	85.43
	5-10	47	13.17
	>10	5	1.40
Blood pressure control status	Controlled	197	55.18
	Uncontrolled	160	44.82
Antihypertensive drugs	One	153	42.86
	Two	145	40.62
	Three and above	59	16.53
Comorbidities	No	260	72.83
	Yes	97	27.17

### Personal factors and social related characteristics of participants

Over half of the study participants (56.02%) were physically active for at least 30 minutes. The majority (84.87%) and (80.67%) of the participants did not consume alcohol, or smoke cigarettes at all, respectively. According to the findings of this study, more than half of the participants (56.30%) had good knowledge about hypertension and its treatment. The majority of study participants (64.43%) had good social support (Table 3).

**Table 3:** Personal factors and social related characteristics of the study participants in selected public hospitals in East Hararghe zone, Eastern Ethiopia, 2023 (n = 357).

Variables	Category	Frequency (N)	Percentage (%)
Physical activity at least 30 minutes of total	Yes	200	56.02
	No	157	43.98
Drinking alcohol	Yes	54	15.13
	No	303	84.87
Smoking cigarette	Yes	69	19.33
	No	288	80.67
Knowledge of HTN	Good	201	56.30
	Poor	156	43.70
Having good social support	Yes	230	64.43
	No	127	35.57

### Organizational related factors of hypertensive patients

The majority of participants (73.67%) reported that drugs were available at hospital pharmacies, and over two-thirds (76.47%) reported having health insurance. Of the participants, 275 (77.03%) had positive relationships with health care professionals, and the majority of respondents (71.71%) reported that the distance between their residence and the health care facility was less than 10 kilometers. For more than half of the respondent’s (54.62%) drugs were changed by the treating doctor. More than half of participants (62.18%) received morning health education on self-management of hypertension (Table 4).

**Table 4:** Organizational and social related factors of adult hypertensive patients in selected public hospitals in East Hararghe zone, Eastern Ethiopia, 2023 (n = 357).

Questions	Category	Frequency (N)	Percentage (%)
Health insurance coverage users	Yes	273	76.47
	No	84	23.53
Drugs available in the hospital pharmacy	Yes	263	73.67
	No	94	26.33
Patient-physician relationship	Good	275	77.03
	Poor	82	22.97
Told by health care providers about importance of taking medications	Yes	290	81.23
	No	67	18.77
Got morning health education about self-management of HTN	Yes	222	62.18
	No	135	37.82
Distance from hospital (in km)	<10	256	71.71
	10-15	62	17.65
	>15	38	10.64
Drug changed by attended doctors	Yes	195	54.62
	No	162	45.38

### Responses of hypertensive patients to MMAS-8 questions

The majority of respondents (66.95%) and (68.07%) said that in the last two weeks had not missed a single day of taking medications or never forgotten to take, respectively. More than two-thirds of respondents (79.55%) said they never missed taking their medication when traveling or leaving home. The majority of respondents (75.91%) did not take their medications the day before their follow-up appointment. More than half of the respondents (57.7%) did not remember taking their medication, and 28.85% stopped taking their medication when their blood pressure was under controlled (Table 5).

**Table 5:** Respondent’s responses to the Morisky medication adherence scale-8 items in selected public hospitals in East Hararghe zone, Eastern Ethiopia, 2023 (n = 357).

Questions	Category	Frequency (N)	Percentage (%)
Do you ever forget to take your medicine?	Yes	114	31.93
	No	243	68.07
In the last two weeks, is there any day when you did not take your high blood pressure medication?	Yes	118	33.05
	No	239	66.95
Have you ever stopped taking your medications or decreased the dose without your doctor order, because you felt worse when you took them?	Yes	73	20.45
	No	284	79.55
Do you forget to take your medications, when you travel or leave the house?	Yes	73	20.45
	No	284	79.55
Did you take your high blood pressure medication yesterday?	Yes	86	24.09
	No	271	75.91
Do you stop taking your medications, when you feel your blood pressure is controlled?	Yes	103	28.85
	No	254	71.15
Have you ever felt distressed for strictly following your high blood pressure treatment?	Yes	104	29.13
	No	253	70.87
How often do you have difficulty to remembering taking all you blood pressure medication?	Yes	151	42.30
	No	206	57.70

### Medication adherence level of respondents

**Fig 1:**
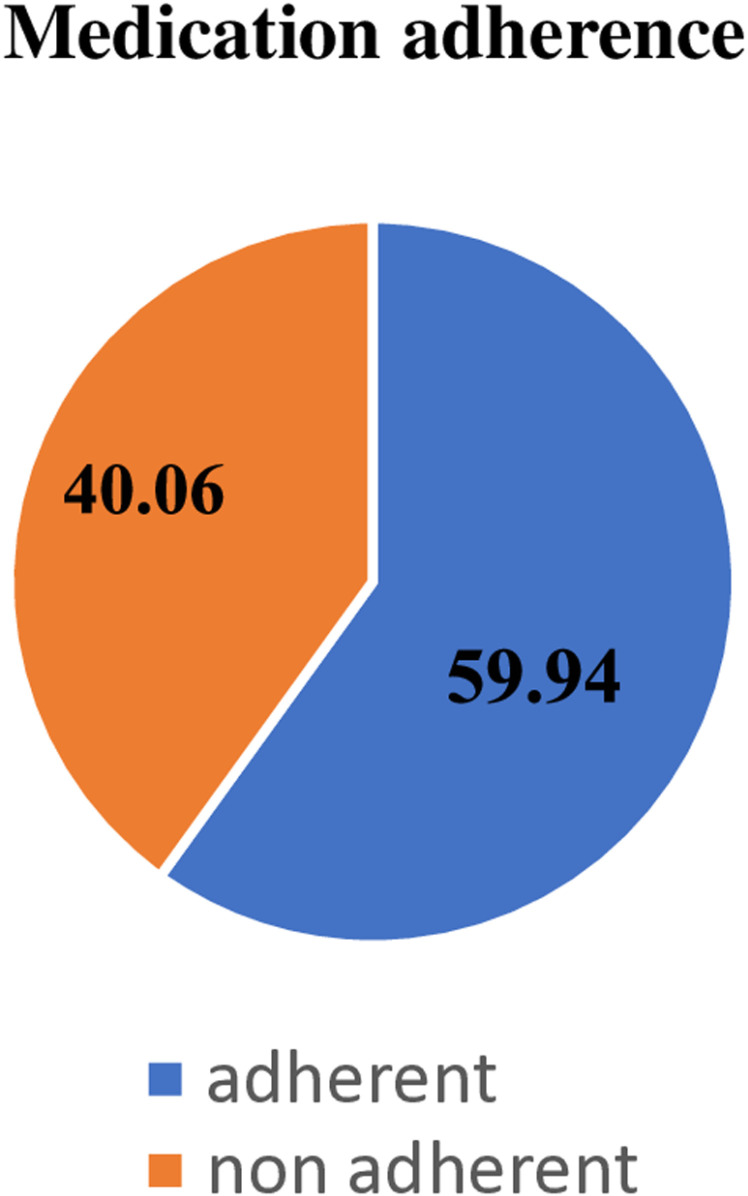
Overall antihypertensive medications adherence among adult hypertensive patients in selected public hospitals in East Hararghe zone, Eastern Ethiopia, 2023 = 357).

The level of adherence to antihypertensive medications was measured using the 8-item Morisky medication adherence scale. The overall level of medication adherence of the respondents was found to be 59.94% (95% CI: 54.65–65.06) (Fig 1**).**

### Factors associated with antihypertensive medication adherence

In multivariable logistic analysis, place of residence, educational status, health insurance coverage, social support, knowledge about hypertension, distance to a health care facility, and number of medications were significantly associated with adherence to antihypertensive medication.

Accordingly, hypertensive patients living in urban area were almost twice as likely to be adherent to their antihypertensive medications (AOR = 1.96; 95% CI: 1.21–3.18)than patients living in rural area. The finding showed that hypertensive patients with a college degree and higher level of education were three times more likely (AOR = 3.41; 95% CI: 1.69–6.87) to adhere to their antihypertensive medications than patients without formal education. Respondents with health insurance coverage and social support were two times (AOR = 2.00; 95% CI: 1.11–3.59) and 1.86 times (AOR = 1.86; 95% CI: 1.13–3.08) more likely to adhere to their antihypertensive medication than their counterparts, respectively. Respondents who were less than 10 kilometers from a health-care facility were 4.6 times more likely to be adherent to their antihypertensive medication (AOR = 4.60; 95% CI: 1.97–10.73) than respondents from more than or equal to 10 kilometers. Respondents who had good knowledge about hypertension and its treatment were 1.75 times (more likely to be adherent to their antihypertensive medication (AOR = 1.75; 95% CI: 1.03–2.97) than their counterparts. Patients taking three or more medications were less likely (AOR = 0.28; 95% CI: 0.12–0.64) to adhere to their medications than those taking fewer than three medications (Table 6).

**Table 6:** Factors associated with adherence to antihypertensive medications among adult hypertensive patients in selected public hospitals in East Hararghe zone, Eastern Ethiopia, 2023 (n = 357).

Variables	Category	Medication Adherence		COR (95% Cl)	AOR (95% Cl)	P-value
		Adherent	Non-adherent			
Residence	Rural	95 (52.78)	85 (47.22)	1	1	
	Urban	119 (67.23)	58 (32.77)	1.83 (1.19-2.81)	1.96 (1.21-3.18)	0.006*
Age (years)	18-39	49 (58.33)	35 (41.67)	1	1	
	40-59	120 (57.14)	90 (42.86)	0.95 (0.57-1.59)	0.82 (0.44-1.51)	0.533
	≥60	45 (71.43)	18 (28.57)	1.78 (0.88-3.58)	1.85 (0.80-4.25)	0.147
Sex	Male	137 (67.16)	67 (32.84)	2.01 (1.31-3.10)	1.70 (0.78-2.74)	0.185
	Female	77 (50.33)	76 (49.67)	1	1	
Educational level	No formal education	56 (49.56)	57 (50.44)	1	1	
	Primary [1–8]	44 (55.0)	36 (45.0)	1.24 (0.70-2.20)	1.20 (0.63-2.27)	0.570
	Secondary [9–12]	43 (59.72)	29 (40.28)	1.50 (0.82-2.74)	1.13 (0.57-2.21)	0.717
	Collage & above	71 (77.17)	21 (22.83)	3.44 (1.86-6.33)	3.41 (1.69-6.87)	0.001*
Comorbidity	Yes	50 (51.55)	47 (48.45)	1	1	
	No	164 (63.08)	96 (36.92)	1.60 (1.00-2.57)	0.71 (0.37-1.36)	0.308
Health insurance users	Yes	174(63.74)	99 (36.26)	1.93 (1.17-3.16)	2.00 (1.11-3.59)	0.020*
	No	40 (47.62)	44 (52.38)	1	1	
Social support	Yes	156 (67.83)	74 (32.17)	2.50 (1.60-3.91)	1.86 (1.13-3.08)	0.015*
	No	58 (45.67)	69 (54.33)	1	1	
Number of medications per day	1	101 (66.01)	52 (33.99)	1	1	
	2	93 (64.14)	52 (35.86)	0.94 (0.58-1.53)	1.20 (0.70-2.06)	0.493
	≥ 3	20 (33.90)	39 (66.10)	0.28 (0.14-0.53)	0.28 (0.12-0.64)	0.003*
Distance from hospital (in km)	< 10	162 (63.28)	94 (36.72)	3.37 (1.80-7.74)	4.60 (1.97-10.73)	0.000*
	10-20	40 (63.49)	23 (36.51)	3.76 (1.60-8.85)	4.09 (1.52-11.03)	0.005
	> 20	12 (31.58)	26 (68.42)	1	1	
Number of children	<5	171 (58.36)	122 (41.64)	1	1	
	≥ 5	43 (67.19)	21 (32.81)	1.46 (0.82-2.58)	1.69 (0.85-3.35)	0.129
Knowledge of HTN	Good Poor	80 (51.28)	76 (48.72)	1.90 (1.23-2.91)	1.75 (1.03-2.97)	0.036*
		134 (66.67)	67 (33.33)	1	1	

* Significant variable at a p-value < 0.05, HTN, Hypertension.

## Discussion

This study was conducted to determine the level of adherence to antihypertensive medication s and its associated factors among adult hypertensive patients in selected public hospitals in East Hararghe Zone, Eastern Ethiopia. Overall adherence to antihypertensive medications was 59.94%. The study identified place of residence, education status, health insurance coverage, social support, knowledge of hypertension, distance toa health care facility, and number of medications as predictors of medication adherence.

The findings of this study showed that the overall level of medication adherence was 59.94% (95% CI: 54.65–65.06). This result is comparable to several reports from Ethiopia, Jimma (61.8%) [19,21], West Gojjam (61.4%) [37], and Debra Berhan (63%) [38]. This comparability might be due to the similar study environment and the similar economic status of the participants. In contrast, this is higher than the findings of studies conducted in Eastern Ethiopia (37%) [8] and Gambia 27% [7]. This difference may be due to the fact that the study in Gambia had a relatively large sample size and was community based; in contrast, the study in eastern Ethiopia was conducted during the COVID-19 pandemic, which is known to impact medication adherence more than at other times. On the other hand finding of this study is lower than the findings of the studies conducted in Northwest Ethiopia (75.1%) [16] and Northwest Ethiopia (67.2%) [20]. The discrepancy may be due to different assessment used applied to assess medication adherence in different population. Another plausible explanation could be the inequality in the educational level of the participants; About 50% of the respondents in this study were not literate.

This study showed that hypertension patients who lived in urban areas were twice as likely to adhere to their medications as patients who lived in rural areas. This is in line with studies conducted in Ethiopia [14,39]. This might be due to the fact that rural residents report several barriers to accessing health care, including transportation difficulties and distance to care, financial constraints, and limited health care facilities [40]. The fact that people living in urban areas have access to different media than people in rural areas may also contribute to disparities in disease awareness.

In the current study, good adherence to antihypertensive medication was higher in patients with college and higher levels of education. This finding is in line with the findings of the studies conducted in Ethiopia [41–43]. This is reported in reverse in the study conducted in Nepal [44]. This might be due to the fact that educated individuals participate more actively in self-monitoring and management plans than their counterparts. Another possible reason might be that an educated person is more likely adhere to their medications because they better understand the costs and benefits of treatment. On the other hand, individuals with low literacy skills and an inability to differentiate between different medications had higher rates of error, non-adherence, and lower medical understanding [45].

The findings of this study showed that respondents who had social support were more likely to adhere to their treatment than those who did not have social support. This finding is similar to the study conducted in central Ethiopia [46]. This might be due to the fact that social support can benefit patients’ health by reducing stress, altering emotional state, improving self-efficacy, and promoting change in unhealthy habits [47]. Furthermore, a patient’s family who provides financial support may remind the patient to take medication.

The findings of this study showed that respondents who had health insurance were twice as likely to be adherent to their medication as respondents who did not have health insurance. This is similar to the findings of the studies conducted in Ethiopia [18,39]. The association might be explained by the fact that the financial burden of paying for drugs was already covered, insurance users didn’t have to worry about their costs. Conversely, non-users of health insurance might not consistently afford to buy their medications while, the cost of medication increases dramatically every day.

The finding of this study revealed that participants who took more than or equal to three medications were less likely to adhere to their medications than participants who took less than three medications. This is similar to the findings of studies conducted in Ethiopia [18,39]. This might be due to the fact that when the number of drugs taken by patients decreases, the complexity of the treatment regimen decreases, so that the patient’s ability to remember and memorize the proper intake of the drugs (the right drugs at the right time) increases. In addition, fewer drugs have fewer adverse effects of treatment and higher drug tolerability, which eventually improve the patient’s adherence status.

The findings of this study also showed that respondents’ knowledge had a positive statistical association with medication adherence. Respondents who had good knowledge about hypertension and its treatment were more likely to adhere to their medication compared to their peers. This finding is comparable to the number of studies conducted in Ethiopia [6,14,39,48]. This is justified on the ground that good knowledge of diseases and their treatment might increase motivation to treatment and self-manage of adverse side effects, which in turn would improve medication adherence. in addition comprehensive disease awareness may encourage patients to participate in the treatment approach actively.

The findings of this study showed a significant association between medication adherence and respondents’ distance from health care facilities. Respondents who came from less than 10 kilometers away were more likely to adhere their medication compared to their peers. This finding is supported by the findings of the studies conducted in northwest Ethiopia [48], and India [49]. This might be because patient who came from a close area are not concerned about transportation fees, vehicles, or the time it takes them to reach the health care facility compared to patients who traveled from a long distance.

### Strengths and limitations

This study looked at the overall magnitude of medication adherence. As part of the study important data about the hypertensive patient was obtained by interviews. Another strength of this study is its increased generalizability, as it conducted at three general hospitals with large populations from both urban and rural areas. This study had the following limitations: First, biases related to self-report, recall, and social desirability may have an impact on the study’s findings. Second, due to the cross-sectional study design and causal conclusion could not be drawn. In addition, patients who did not visit hospitals during the data collection period and private hospitals were not included in this study.

### Conclusions

The magnitude of medication adherence was found to be very low compared to WHO standards. Medication adherence is statistically significantly associated with place of residence, educational status, health insurance coverage, social support, knowledge of hypertension, distance to a health care facility, and number of medications. To improve patients’ adherence status, the government and health bureau should improve the accessibility of health care facilities and strengthen health insurance coverage. In addition, health care professionals needto simplify treatment plans and educate patients about hypertension and its treatment.

## Abbreviations

AOR: Adjusted Odds Ratio
BMI: Body Mass Index
BP: Blood Pressure
CAD: Coronary Artery Disease
CI: Confidence Interval
COR: Crude Odds Ratio
CVD: Cardio-vascular disease
HTN: Hypertension
MMAS: Morisky’s Medication Adherence Scale
WHO: World Health Organization
